# Who stays, who drops out? Biosocial predictors of longer-term adherence in participants attending an exercise referral scheme in the UK

**DOI:** 10.1186/1471-2458-12-347

**Published:** 2012-05-11

**Authors:** Patrick Tobi, Emee Vida Estacio, Ge Yu, Adrian Renton, Nena Foster

**Affiliations:** 1 Institute for Health and Human Development, University of East London, London, E15 4LZ, UK; 2 School of Psychology, Keele University, Staffordshire, ST5 5BG, UK; 3 School of Health and Bioscience, University of East London, London, E15 4LZ, UK

**Keywords:** Attendance, Biosocial, Exercise referral, Longer term adherence

## Abstract

**Background:**

Exercise referral schemes are one of the most popular forms of physical activity intervention in primary care in the UK and present an opportunity to better understand the factors related to exercise adherence. But standard schemes tend to be delivered over a short period and so provide information about the factors associated with short-term adherence. This retrospective register-based study of a longer-duration scheme allowed investigation of longer-term adherence.

**Methods:**

Social, physiological and anthropometric data were extracted from records of a cohort of ERS participants who had enrolled between 01 January and 31 December 2007 (n = 701). Characteristics of adherers and non-adherers were compared and potential predictors of longer-term adherence examined using binomial logistic regression.

**Results:**

Significant adjusted odds ratios predicting longer-term adherence were found for age and medical condition. For every 10 year increase in age, the odds of people continuing exercise increased by 21.8% (OR = 1.02; CI = 1.00 to 1.04; p = 0.03). Participants referred with orthopaedic (OR = 0.25; CI = 0.07-0.94; p = 0.04), cardiovascular (OR = 0.18; CI = 0.05-0.70; p = 0.01) and other (OR = 0.20; CI = 0.04-0.93; p = 0.04) problems had significantly lower odds of adhering than those with metabolic conditions.

**Conclusion:**

Improved understanding of the factors that influence adherence to exercise referral schemes will enable providers develop better referral guidance and tailor schemes to better meet participants’ needs. Longer-term schemes offer the opportunity to understand participants’ likelihood of maintaining adherence to exercise.

## Background

Since the early 1990s there has been significant and sustained growth in the number of exercise referral schemes (ERS) in the UK [1,2]. By 2005, 89% of primary care organisations in England ran an ERS making it one of the most common forms of physical activity intervention in primary care [3]. Exercise referral is the practice of referring a person from primary care to a qualified exercise professional who uses relevant medical information about the person to develop a tailored programme of physical activity usually lasting from 10–12 weeks [1]. The objective is to increase opportunities for exercise by providing access to facilities and activities in the notion that it will encourage long-term exercise behaviour [4]. Schemes typically operate as a partnership between the local authority, Primary Care Trust (PCT) and private leisure service providers.

Despite a national review that cast doubt on their effectiveness in the longer term (i.e. >12 weeks) [5], government policy continues to encourage exercise referral therapy in appropriate circumstances, such as supporting the medical management of conditions [6]. But many schemes experience poor rates of attendance, a factor that may contribute to their inability to demonstrate effectiveness [7,8]. A systematic review in 2005 reported that 80% of participants dropped out before completion [9]. Some studies have examined user (self motivation, socio-demographic) and service (source of referral, design) characteristics of the schemes to explain attendance levels. However, the schemes themselves seldom last more than 12 weeks, meaning that they are only able to provide information about factors related to short-term exercise adherence. These factors cannot automatically be assumed to be the same ones that influence longer-term adherence to exercise [10].

Another reason the evidence base largely focuses on short-term behaviour is because schemes lack the capacity to monitor participants’ subsequent transition to mainstream gym or leisure centre activities. This is often because of poor record keeping or the use of unlinked data capture systems making it difficult to track individuals across programmes even when they take place within the same leisure facility. Even then, the extent to which leisure centre attendance is a valid proxy measure of exercise adherence is debatable. As a result, longer-term exercise behaviour, and the factors that influence it, remains an area that needs to be illuminated [11].

This paper reports findings from the quantitative component of a mixed methods evaluation of an exercise referral scheme in the London Borough of Greenwich which used a combination of qualitative and quantitative approaches to analyse participants exercise behaviour, experiences and barriers to participation in the scheme [12]. The relatively high adherence rates - 58% at 13 weeks and 45% at 20–26 weeks compared to schemes elsewhere, and the socio-cultural and clinical diversity of participants made it a particularly appropriate site to investigate the factors linked to exercise adherence over the longer term.

## Methods

### Setting

Healthwise ERS evolved from a partnership between NHS Greenwich, the Local Authority and Greenwich Leisure Limited, a major provider of public leisure services. It commenced in 2005 as a subsidised programme for adult local residents with existing health conditions or at risk of developing conditions where physical activity could be of benefit. Black and Minority Ethnic (BME) people were particularly targeted. Participants were referred by their general practitioner (GP) to leisure centres in the borough where they attended motivational and educational classes and accessed a range of classes and courses designed to help them manage and improve their condition. The classes included British Association for Cardiac Rehabilitation (BACR) phase IV cardiac rehabilitation classes, gym based supervised sessions, swimming and water workouts, circuit training, healthy walks, weight management courses and group exercise options. Sessions were supervised by Healthwise facilitators who held a minimum of Register of Exercise Professionals (REPS) level 3 qualification as well as a GP referral qualification that enabled them work with patients with a range of medical conditions. The majority of sessions lasted 1 hour (range 45 to 90 minutes) and participants usually attended a minimum of 2 or 3 sessions a week. The total number of sessions attended varied depending on the exercise plan agreed with each participant and attendance was calculated on this basis. Physical and physiological assessments were made at enrolment and then at the 7th, 13th and 20-26th weeks.

### Definitions

Terminology used by ERS researchers to describe aspects of participation in the schemes, while fairly similar, has not always been uniformly applied. Uptake refers to initial attendance, take up or enrolment. Attendance describes subsequent continuation after take up. Related to this is adherence which denotes the level and duration of participation. It is usually qualified as early, long-term or very long-term and typically described in terms of completers or adherers (participants who successfully finish the prescribed number of exercise sessions) and non-completers or non-adherers (those who drop out, do not attend a pre-specified minimum number of sessions – usually 70-80%, or fail assessments for any other reason). We used the phrase ‘longer-term adherence’ in this study to reflect both the unusual duration of the scheme and length of participation. This is consistent with the recommendation that ERS providers need to clearly define what they mean by the terms they use in order to provide more accurate and meaningful evaluation data [1].

### Participants

**Figure 1:**
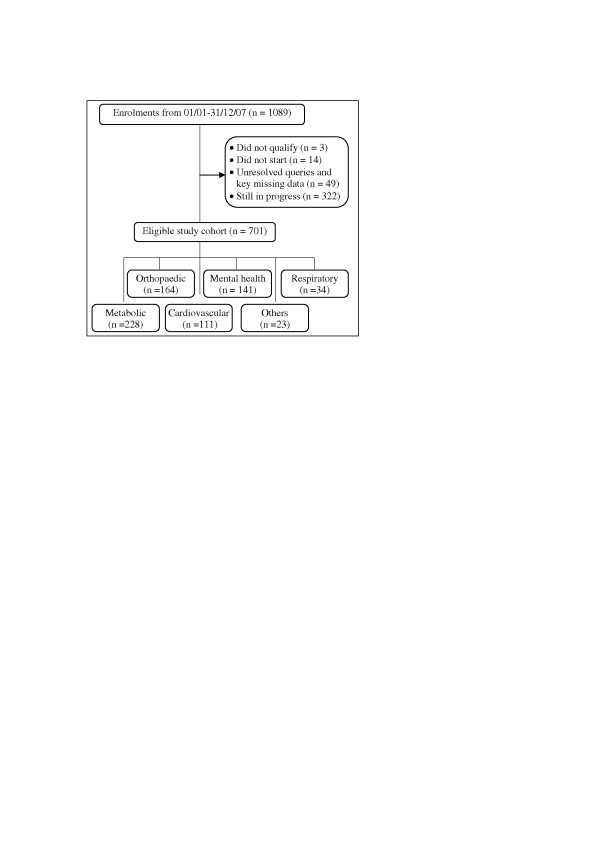
Study cohort.

We identified from the Healthwise database an initial population of 1089 participants enrolled on the scheme during the study period, from which a final cohort of 701 was eligible for analysis after excluding cases that were still in progress at the time and had not yet had a final assessment (n = 322) or were disqualified for other reasons such as not meeting the eligibility criteria, failing to turn up and missing data on key variables (n = 66). Participants’ primary referral diagnosis was coded into one of 6 clinical categories (Figure 1). No information was available on how many patients had declined the offer of referral by their GP. The study was approved by the University of East London Research Ethics Committee (ETH/09/01) and access to the data facilitated by the study funders.

### Measures

#### Primary outcome measure

Longer-term adherence was the primary outcome measure and was measured by successful assessments at both the 13th and 20-26th weeks. Unlike prior definitions that relied only on attendance at the final session, this is a more rigorous definition of adherence that takes account of both the duration and frequency of attendance. It resolves the contentious status of participants whose attendance over the course of the programme is poor but still turn up for the final assessment. On this basis we separated the cohort into two groups: adherers and non-adherers. The latter were people who did not undergo, or failed at least one of the assessments. Failure was determined as attendance at <80% of scheduled sessions. The level was based on adherence data from earlier research [9]. The 13th week assessment corresponded broadly to the 10–12 weeks duration of other similar schemes and so allowed comparison with these.

#### Exposure measures

We extracted social, physiological and anthropometric records on the 701 eligible participants who had joined the scheme between 01 January and 31 December 2007. The 12 month time frame allowed the effects of seasonal variation in physical activity levels and physiological responses to exercise to be taken into account [13]. Data included age, gender, ethnicity, postcode, occupational group, method of payment, medical referral category, height and weight at enrolment. Baseline BMIs were computed from height and weight data and categorised as healthy (18.5–24.9 kg/m2) or unhealthy (i.e. overweight/obese; ≥25 kg/m2) using national criteria [14]. Systolic blood pressure (SBP) of ≥140 mmHg and/or diastolic blood pressure (DBP) of ≥90 mmHg and respectively at enrolment were used to identify hypertensive individuals [15]. Although able to return more statistically powerful analysis in a continuous form, we analysed BP dichotomously to enable discussion of cut off values that are meaningful and commonly applied in policy and practice.

Deprivation information was also examined in order to control for its effects. Each participant was assigned an Index of Multiple Deprivation (IMD) score at the Lower Super Output Area (LSOA) level based on their address postcode. The IMD is a summary measure of area-level deprivation that combines weighted scores in seven deprivation domains – income; employment; health and disability; education, skills and training; living environment; crime; and barriers to housing and services. LSOAs are small areas across the country of about 1500 people that, unlike electoral wards, are not subject to boundary changes and so improve the reporting and comparison of area-level statistics [16]. Each of the LSOAs in England and Wales is given a ranked score from 1 (most deprived) to 32,482 (least deprived). The ranks were grouped into IMD national reference quintiles with 1 representing the most deprived areas.

### Data analysis

Frequency distribution histograms and box plots were visually inspected to check normality of continuous variables (age, IMD, BP and BMI). Uni- and bivariable descriptive statistics were used to characterise the cohort, explore associations between variables and differences between the groups. The chi-squared statistic was used for categorical variables and t- test for continuous ones. Given the dichotomous nature of exercise adherence, binomial logistic regression was used to estimate the effects of predictors. Candidate variables with the highest potential predictive power were chosen for model building based on p-values in unadjusted analyses showing evidence of a relationship at the 20% level and on consistent associations identified in other studies [17]. Multicollinearity was excluded using a value of greater than 0.90 to identify highly correlated variables that could result in unstable estimates [18]. Continuous variables were first centred before using General Linear Models to test interaction terms between age, gender, BP and BMI in 2- and 3-variable combinations [19]. The only near-significant interaction was found between gender and BP and this was at the 6% level (*F*(1,694) = 3.493, *p* = 0.062), so we did not include any interaction terms in the final regression model. Predictors were fitted simultaneously into the model with age examined as a continuous variable and all others as categorical. The Hosmer-Lemeshow test showed evidence of good model fit with the data (p > 0.05). All analysis was carried out with SPSS Version 15.0 for Windows (SPSS, Inc., Chicago, IL).

## Results

### Characteristics of participants

Longer-term exercise adherence was relatively high with 45% of participants attending more than 80% of scheduled sessions over the full duration of 20–26 weeks (Table 1). At the 13th week when most other schemes terminate, the completion rate was 58% (data not shown). Across the whole cohort, the proportion of women enrolled (60%) was comparable to schemes elsewhere (60-65%) but the mean age was lower (46 years versus 51 years) [20-22]. As with other schemes most participants were White, but the proportion of people from a Black and Minority Ethnic (BME) background was higher than in the general population of Greenwich (37% versus 29%) [23] reflecting the success of the scheme’s policy to target BME groups who suffer worse health inequalities.

**Table 1:** Profile of participants by adherence status

**Characteristics**	**Adherers (%)**		**Non-adherers (%)**		***p***
n (%)	314	(45.0)	387	(55.0)	-
Gender					0.619^a^
Men (%)	124	(40.0)	160	(41.0)	
Women (%)	190	(60.0)	227	(59.0)	
Mean age (SD)	48.4	(14.5)	44.4	(13.2)	<0.001^b^
Ethnic group					0.856^a^
White	195	(63.1)	238	(63.4)	
Black	62	(20.1)	82	(21.9)	
Asian	35	(11.3)	37	(9.9)	
Other non-White	17	(5.5)	18	(4.8)	
Occupational group					0.002^a^
Unemployed	97	(33.0)	148	(43.2)	
Retired	69	(23.5)	46	(13.5)	
Unskilled/partly skilled	30	(10.2)	27	(7.9)	
Skilled	71	(24.1)	76	(22.2)	
Managerial/professional	27	(9.2)	45	(13.2)	
Mean IMD score (Range)	35.4	(4.4-61.5)	35.9	(12.8-61.5)	0.591^b^
Method of payment					0.153^a^
Long term (monthly/direct debit)	229	(87.7)	188	(83.2)	
Short term (pay & play)	32	(12.3)	38	(16.8)	
Clinical category					0.282^b^
Metabolic	103	(32.8)	125	(32.3)	
Orthopaedic	78	(24.8)	86	(22.2)	
Mental health	53	(16.9)	88	(22.7)	
Cardiovascular	52	(16.6)	59	(15.2)	
Respiratory	14	(4.5)	20	(5.2)	
Other	14	(4.5)	9	(3.2)	
Mean BMI at baseline (SD)	31.9	(6.8)	32.2	(7.2)	0.630^a^
Baseline blood pressure					0.389^a^
Normal	175	(56.1)	229	(59.3)	
Elevated	137	(43.9)	157	(40.7)	

^a^Chi squared test, ^b^T test.

Nearly four in 10 participants were unemployed and a further two in 10 were retired. A high proportion of participants were in poor physical health at enrolment as measured by high blood pressure and mean BMI at baseline. Eighty four percent of participants had an unhealthy BMI of which 26% were classified as overweight and 58% as obese (breakdown not shown). Four clinical categories –metabolic, orthopaedic, mental health and cardiovascular – were responsible for over 90% of referrals to the scheme.

At sub-cohort level, the age profile and occupational backgrounds of participants differed significantly. The mean age of adherers was higher as a result of a greater proportion of them being over 50 years (43.1% to 31.9%, age category data not shown). A greater proportion of people who adhered were retired and a lower proportion were unemployed or held managerial/professional jobs compared to non-adherers.

Results of the multivariable analysis are presented in Table 2. The adjusted odds ratios showed that participants’ age and clinical condition were independently predictive of longer-term adherence. For every 10 year increase in age, the odds of people adhering to the scheme increased by 21.8% (OR = 1.02, CI = 1.00 to 1.04; p = 0.03). Participants referred with orthopaedic (OR = 0.25, CI = 0.07 to 0.94, p = 0.04), cardiovascular (OR = 0.18, CI = 0.05 to 0.70; p = 0.01) and other problems (OR = 0.20, CI = 0.04 to 0.93; p = 0.04) had significantly lower odds of adhering than those with metabolic conditions.

**Table 2:** Adjusted odds ratios for longer-term exercise adherence

**Covariate**	**OR**	**95% CI**	***p***
Age (continuous)	1.02	1.00-1.04	0.03
Occupation			0.47
Unemployed	1.00 (ref)		
Retired	1.53	0.81-2.87	0.19
Unskilled/partly skilled	1.75	0.78-3.93	0.17
Skilled	1.79	0.74-4.32	0.19
Managerial/professional	1.86	0.95-3.65	0.07
Method of payment			
Direct debit	1.00 (ref)		
Cash	0.58	0.32-1.05	0.07
Clinical category			0.10
Metabolic	1.00 (ref)		
Orthopaedic	0.25	0.07-0.94	0.04
Mental health	0.32	0.08-1.23	0.10
Cardiovascular	0.18	0.05-0.70	0.01
Respiratory	0.33	0.08-1.32	0.12
Other	0.20	0.04-0.93	0.04

OR = odds ratio; CI = confidence interval; ref = reference category.

## Discussion

Few studies investigating the determinants of adherence in exercise referral schemes have been conducted on programmes lasting longer than 12 weeks, and so what is known largely relates to short-term exercise behaviour. This study investigated a programme whose duration (double the length of most other schemes at 20–26 weeks) and comparatively high adherence rates (45% at 20–26 weeks) offered a unique opportunity to gain a deeper understanding of some of the factors that influence exercise behaviour over longer periods of time. Allowing for heterogeneity in the nature and quality of schemes, it also enabled some insight into which adherence factors remain consistently influential or come into play at a later stage. A further feature of the Healthwise scheme was the size and diversity of the participant pool referred for exercise which allowed robust numbers to be generated in the different categories of predictor variables and so offered the statistical power necessary to deliver meaningful information on the outcome measure.

## Comparison with existing literature

In the general population, physical activity reduces with age [24,25] and people over the age of 50 represent the most sedentary segment of the adult population [26]. But with exercise referral the reverse seems to be the case and our finding of an association between older age and adherence in this scheme concurs with other studies [7,9,17,27]. A number of factors are thought to explain this – older people are less time-constrained, more likely to value the social interaction offered by the group based approaches, and may find it easier to incorporate the scheme exercise activities (such as walking, swimming and cycling) into their everyday life. Whatever the reasons, the finding suggests that age remains a consistent predictor of adherence over both the short and longer term. Other evidence from a study in the Netherlands among older (>50 years) participants found that the occurrence and duration of lapses in attendance, the intention to continue participation, the perceived quality of the exercise programme, and baseline attitude were also important factors in the maintenance of exercise participation [10].

With other social determinants such as gender, ethnicity and deprivation, effects on exercise referral uptake and attendance have yet to be firmly established. In some studies ethnicity was not reported [7], while in others the effect of socioeconomic deprivation on referral uptake was inconsistent [7,20,22]. One explanation for the weak evidence may be that schemes operate in different social and environmental contexts and so are not strictly comparable. Our study did not find these factors to have any impact on longer-term adherence.

Another association we identified was with clinical condition. Compared to people with metabolic conditions (diabetes, hyperlipidemia, obesity, hyperthyroidism and hypothyroidism) who were the largest group and used as the reference category, the odds of longer-term adherence were significantly lower for participants referred with orthopaedic (arthritis, back pain, osteoporosis, fibromyalgia and other bone/musculoskeletal disorders), cardiovascular (myocardial infarction, coronary artery bypass graft surgery and coronary angioplasty, angina and silent ischemia, atrial fibrillation, chronic heart failure, peripheral arterial disease and hypertension) and other conditions (neuromuscular, sensory, miscellaneous complaints). Research investigating the association between the clinical reason for referral and attendance at ERS has highlighted higher rates of attendance in participants with serious cardiovascular conditions [28] and lower rates in people who were overweight/obese or had a respiratory or mental health condition [17,21,29]. Functional limitation, perceived seriousness of the problem and low self motivation are among the explanations underpinning differing rates of adherence in these groups.

Although our interest was in biosocial explanations for exercise adherence behaviour, the design features of schemes have also been demonstrated to influence adherence. Research has highlighted the link between higher attendance and the flexibility of the scheme, activities tailored to participants’ interests and capabilities, convenient timing, friendly and supportive staff, a wide range of activities, ‘break’ periods and activities that fit easily into everyday life such as such as walking, swimming and cycling [4,30,31]. Characteristics of the referrer are also important as participants referred from cardiac and practice nurses had higher levels of adherence than participants referred by general practitioners [32].

## Study limitations

Owing to data limitations, an inherent weakness of this study was our inability to simultaneously analyse and draw links between factors operating across all components of the scheme i.e. the participants, the programme and the context of implementation. This would have provided a more granular understanding of adherence and how participant factors are mediated or moderated by the other scheme components, more so if the providers had elaborated the health behaviour change theory underpinning the scheme.

Our assessment of exercise adherence relied solely on the monitoring data collected by the scheme provider. It was therefore sensitive to data entry errors and missing data, even though as much effort as possible was made to recover and input missing information and clean the database. Although studies have attempted to define successful adherence to exercise on the basis of the proportion of scheduled sessions attended, evidence about the level of participation necessary to deliver health benefits is still uncertain and further compounded by the lack of detailed information about the specific activities provided. The low number of socio-demographic variables available for analysis limited the amount of information that could be gleaned. The marital status of participants was not stated although it is known that people who are married or have a partner are more likely to maintain attendance [33]. Also, we had no data on levels of physical activity and motivation at baseline which can influence adherence at later stages. Inadequate participant profiling is a major deficit in many schemes [9] and owes to inadequate guidance on the collection of monitoring data as well as poor evaluation culture.

While it was not a major concern in our study, the high dropout rate experienced by many exercise schemes has also raised questions about the appropriateness of referrals. As a result, Johnston and colleagues recommended that existing referral guidelines be broadened to take into consideration the medical conditions suitable for referral as well as the stage of readiness of the patient to take up the referral [34]. Poor record keeping and the use of unlinked data systems by many schemes make it often impossible to accurately track participants’ subsequent attendance at the gym or leisure centre after they have completed the exercise referral programme. This scheme was no different to others in that respect and meant that our determination of longer-term adherence was predicated on behaviour only within the scheme. Some participants who dropped out may have continued exercise on their own or at the gym or another facility elsewhere. Therefore, our study only offered insight into people’s adherence patterns within a formal exercise programme, even if one of long duration. The lack of information about the subsequent exercise behaviour of non-completers has been highlighted [35] and is a priority area for further research.

## Conclusions

The findings from this scheme have shown some similarities but also differences to other largely shorter-term schemes. This might indicate a shift in adherence factors as participants’ progress from early to later stages of exercise adherence; although it is not possible to be certain without more comprehensive analysis of data across all aspects of the scheme internally and externally, and analysis of other long-term schemes. But it may challenge the view that the factors that influence people’s attendance during the scheme are likely to be similar to those that exert an influence after the scheme.

While short schemes can inform about uptake and attendance influences, longer-term schemes may provide an understanding of maintenance (or adherence) influences and might be more predictive of post-completion exercise behaviour. The information can help clinicians and providers in improving referral guidance and the design of exercise activities so that participants are given appropriately tailored support.

The limited guidelines developed by the National Quality Assurance Framework [36] in 2001 have now been addressed more comprehensively by the British Heart Foundation exercise referral toolkit [1] and should hopefully help providers develop data systems to reliably capture information about the aspects of exercise behaviour where not much is known including longer-term adherence, post-drop out and post-completion exercise behaviour.

## Competing interests

PT and EE were part of a team commissioned to evaluate Healthwise.

## Authors’ contributions

PT designed the analysis, wrote the manuscript, and carried out statistical analysis with assistance from AR and GY. EE was co-responsible for data collection, design and coordination of the study. NF was involved in interpretation and implications of the analysis. All authors contributed to developing the manuscript, and read and approved the final version.

## Pre-publication history

The pre-publication history for this paper can be accessed here:

http://www.biomedcentral.com/1471-2458/12/347/prepub

## References

[B1] British Heart Foundation National Centre A toolkit for the design, implementation and evaluation of exercise referral schemes. Guidance for referring health professionals, exercise referral professionals and exercise referral scheme commissioners [ http://www.bhfactive.org.uk/sites/Exercise-Referral-Toolkit/]

[B2] Labour Research Department Exercise on prescription: a report for the Chartered Society of Physiotherapy 2004 London

[B3] Sowden SL Raine R Running along parallel lines: how political reality impedes the evaluation of public health interventions. A case study of exercise referral schemes in England J Epidemiol Community Health 2008 62 835 841 10.1136/jech.2007.069781 18701737

[B4] Thurston M Green K Adherence to exercise in later life: how can exercise on prescription programmes be made more effective? Health Promot Int 2004 19 379 387 10.1093/heapro/dah311 15306622

[B5] NICE A rapid review of the effectiveness of exercise referral schemes to promote physical activity in adults 2006. Public Health Collaborating Centre for Physical Activity, National Institute for Health and Clinical Excellence.

[B6] Department of Health DH statement on exercise referral 2007 London: Department of Health

[B7] Gidlow C Johnston LH Crone D Morris C Smith A Foster C Socio-demographic patterning of referral, uptake and attendance in Physical Activity Referral Schemes J Public Health 2007 29 107 113 10.1093/pubmed/fdm002 17341508

[B8] Williams NH Hendry M France B Lewis R Wilkinson C Effectiveness of exercise-referral schemes to promote physical activity in adults: systematic review Br J Gen Pract 2007 57 979 986 10.3399/096016407782604866 18252074 PMC2084138

[B9] Gidlow C Johnston LH Crone D James D Attendance of exercise referral schemes in the UK: A systematic review Heal Educ J 2005 64 168 186 10.1177/001789690506400208

[B10] Stiggelbout M Hopman-Rock M Crone M Lechner L van Mechelen W Predicting older adults' maintenance in exercise participation using an integrated social psychological model Heal Educ Res 2006 21 1 14 10.1093/her/cyl147 15980075

[B11] Sørensen J Sørensen JB Skovgaard T Bredahl T Puggaard L Exercise on prescription: changes in physical activity and health-related quality of life in five Danish programmes The European Journal of Public Health 2011 21 56 62 10.1093/eurpub/ckq003 20371500

[B12] Tobi P Estacio EV Seesaghur A Nabingi S Cawley J Evaluation of Healthwise exercise referral scheme (Final Report). Prepared for Greenwich Teaching Primary Care Trust and Greenwich Leisure Limited 2009 University of East London: Institute for Health and Human Development

[B13] Atkinson G Drust B Seasonal Rhythms and Exercise Clinics in Sports Medicine 2005 24 e25 e34 10.1016/j.csm.2004.11.001 15892915

[B14] NICE Obesity: guidance on the prevention, identification, assessment and management of overweight and obesity in adults and children NICE clinical guideline 43. 2005. London, National Institute for Health and Clinical Excellence. 22497033

[B15] NICE Hypertension: management of hypertension in adults in primary care (partial update of NICE clinical guideline 18) NICE clinical guideline 34. 2006. London, National Institute for Health and Clinical Excellence

[B16] Office of the Deputy Prime Minister The English Indices of Deprivation 2004 2004. London Stationary Office.

[B17] James DV Johnston LH Crone D Sidford AH Gidlow C Morris C Factors associated with physical activity referral uptake and participation J Sports Sci 2008 26 217 224 10.1080/02640410701468863 17943595

[B18] Tabachnick B Fidell L Using multivariate statistics 2001 4 MA: Allyn and Bacon, Boston

[B19] Judd CM Kenny DA McClelland GH Estimating and testing mediation and moderation in within-participant designs Psychological Methods 2001 6 115 134 11411437 10.1037/1082-989x.6.2.115

[B20] Harrison RA McNair F Dugdill L Access to exercise referral schemes - a population based analysis J Public Health 2005 27 326 330 10.1093/pubmed/fdi048 16207736

[B21] James D Mills H Crone D Johnston LH Morris C Gidlow CJ Factors associated with physical activity referral completion and health outcomes J Sports Sci 2009 27 1007 1017 10.1080/02640410903214248 19847684

[B22] Sowden SL Breeze E Barber J Raine R Do general practices provide equitable access to physical activity interventions? Br J Gen Pract 2008 58 e1 e8 18826774 10.3399/bjgp08X342237 PMC2553553

[B23] Greenwich Teaching Primary Care Trust Building health into the future: a report on health inequalities in greenwich. the annual report of the director of public health 2008 2008 London: Directorate of Public Health, Greenwich TPCT

[B24] Craig R Mindell J Hirani V The Health Survey for England 2008: Physical activity and fitness 2009 London: NHS Information Centre for Health and Social Care

[B25] Department of Health Choosing Health? Choosing activity: a consultation on how to increase physical activity 2004 London: he Stationery Office

[B26] BHF National Centre Guidelines on the promotion of physical activity with older people 2008 British Heart Foundation National Centre for Physical Activity and Health: Loughborough University

[B27] Morgan O Approaches to increase physical activity: reviewing the evidence for exercise referral schemes Public Health 2005 119 361 370 10.1016/j.puhe.2004.06.008 15780323

[B28] Dugdill L Graham R Promoting physical activity: Building sustainable interventions 2004 Oxford: Blackwell Science Publishing 240 255

[B29] Crone D Johnston LH Gidlow C Henley C James DVB Uptake and participation in physical activity referral schemes in the UK: an investigation of patients referred with mental health problems Issues in Mental Health Nursing 2008 29 1088 1097 10.1080/01612840802319837 18853348

[B30] Martin C Woolf-May K The retrospective evaluation of a general practitioner exercise prescription programme Journal of Human Nutrition and Diet 1999 12 suppl 1 32 42

[B31] Wormald H Ingle L GP exercise referral schemes: Improving the patient's experience Heal Educ J 2004 63 362 373 10.1177/001789690406300407

[B32] Dugdill L Graham RC McNair F Exercise referral: the public health panacea for physical activity promotion? A critical perspective of exercise referral schemes; their development and evaluation Ergonomics 2005 48 1390 1410 10.1080/00140130500101544 16338708

[B33] Lord JC Green F Exercise on prescription: does it work? Heal Educ J 1995 54 453 464 10.1177/001789699505400408

[B34] Johnston LH Warwick J De Ste CM Crone D Sldford A The nature of all 'inappropriate referrals' made to a countywide physical activity referral scheme: Implications for practice Heal Educ J 2005 64 58 69 10.1177/001789690506400107

[B35] Sørensen JB Skovgaard T Puggaard L Exercise on prescription in general practice: A systematic review Scand J Prim Health Care 2006 24 69 74 10.1080/02813430600700027 16690553

[B36] Department of Health Exercise referral systems: a national quality assurance framework 2001 London: Department of Health

